# Effect of vein-bionic surface textures on the tribological behavior of cylindrical roller thrust bearing under starved lubrication

**DOI:** 10.1038/s41598-021-00800-x

**Published:** 2021-10-28

**Authors:** Risheng Long, Chao Zhao, Yimin Zhang, Yibing Wang, Yueyong Wang

**Affiliations:** 1 Equipment Reliability Institute, Shenyang University of Chemical Technology, Shenyang, 110142 China; 2 School of Mechanical Engineering, Shenyang University of Technology, Shenyang, 110870 China

**Keywords:** Mechanical engineering, Surface patterning

## Abstract

To reveal the effect of vein-bionic surface textures on the tribological behavior of cylindrical roller thrust bearings (CRTBs) under starved lubrication, six kinds of leaves (Forsythia, Clausena lansiu, Ash, Purple leaf plum, Pipal and Apricot) were chose and their simplified patterns were fabricated on the shaft washers of CRTBs using laser surface texturing. The coefficients of friction (COFs) of vein-bionic textured bearings were measured using a vertical universal wear test rig. Their mass losses and worn surfaces were also characterized. The results show that: There is important influence of the symmetry of vein-bionic textures and the number of secondary veins on the friction and wear properties of vein-bionic textured CRTBs under starved lubrication. Compared to the smooth group, the COFs and mass losses of vein-bionic textured bearings are all reduced. Among all groups, the tribological performance of bearings with a pattern inspired from Ash is the best. Its wear loss is reduced by 16.23% and its COF is reduced by 15.79%. This work would provide a valuable reference for the raceway design and optimization of roller rolling element bearings.

## Introduction

For all electromechanical system or industrial equipment, the relative movements between adjacent parts are inevitable and necessary. Consequently, as one of the main reasons for the failure of mechanical parts, the friction and wear between moving parts is undesirable and also unavoidable, resulting in a large amount of power consumption and material losses. To meet the continuously increasing demands for higher energy efficiency, stronger load carrying capacity and longer service life, it is of great significance to lower such losses^1,2^. Laser surface texture (LST) is commonly used to create some macro-/micro units, e.g. pillar, pit, groove and grid, distributed in a periodic geometry or pattern on the target surface (in whole or in part) of different materials (e.g. metallic materials, ceramics^3^, polymers^4^), and has proved to be one of the most cost-effective way to tune the friction and wear properties of various mechanical components^5–7^, including sliding bearing^8^, journal bearing^9^, piston rings^10^, gears^11^, under different lubrication regimes^12–14^. The main mechanisms of friction-reducing and wear-improvement of textured parts include: increase of oil film thickness, reducing of effective contact area, storage of debris and secondary lubrication^5,12,15^.

The effect of surface textures on the tribological behavior of sliding/rolling tribo-pairs mainly depend on their pattern parameters (e.g. unit shape, area ratio, distance of adjacent units) and texture dimensions (e.g. depth, diameter, width, ratio of depth-diameter) as well as lubrication regimes^16–20^. Besides those simple, regular and uniformly arranged isotropic ones, some new textures have also been studied. Inspired by snake, cytister bengalensis aube and golf ball, Wang et al. designed three texture patterns and studied their friction and wear properties under seawater lubrication^14^. Atwal et al. investigated the tribological behaviors of a hydrodynamic thrust bearing with microrectangular pocket and Labeo rohita fish scale texture^21^. Lu et al. designed the irregular gourd-shaped surface texture and researched the tribological performance of gourd-shaped textured stainless steel using an UMT2 tester^22^. Li et al. investigated the dry friction behaviors and wear mechanisms of Al_2_O_3_/TiC ceramics with biomimetic shark skin surface texture^23^. Some multi-scale surface patterns were also researched^6^.

The vein of leaf is a natural anisotropic non-smooth surface, which has selectively evolved in nature for millions of years. The experimental and finite element simulation results of the model based on original leaf show that the vein structure has good mechanical properties and can maintain small internal strain energy^24^. However, the publication about the research of vein-bionic surface in surface texture field is still blank, especially the influence of vein-bionic textures on the tribological behavior of rolling element bearings (REBs). REB may be cheap, but its failure will be costly. To reduce the huge energy waste and material losses caused by the friction and wear between the moving parts (e.g. washers, rollers and cage), it is of great significance to improve the wear resistance of REBs, and prolong their mild wear periods^1,5^.

Therefore, the cylindrical roller thrust bearings (CRTBs) were chose in this work to reveal the effect of vein-bionic textures on the tribological behavior of REBs, due to its separability and easy machinability through LST method^25–27^. The leaves of six kinds of plants (Forsythia, Clausena lansiu, Ash, Purple leaf plum, Pipal and Apricot) were randomly collected in the campus of Shenyang University of Chemical Technology, and six vein-bionic textures were designed and marked on the shaft washers of 81107TN bearings (YFB, Changzhou, China) by a fiber laser marking system. The friction and wear properties of bearings were researched using a vertical universal tribo-meter (MMW-1A, Jinan Huaxing, China) under starved lubrication with a customized tribo-pair. The wear losses and worn surfaces were characterized by an electronic analytical balance (EX225D, Ohaus) and a 3D surface profilometer (VK-1050, Keyence, Japan), respectively. The influence of vein-bionic patterns on the tribological properties of CRTBs was briefly discussed. This work would provide a valuable reference for the raceway design and optimization of REBs.

## Designing, preparation and setting

### Designing and preparation

81107TN bearing was used in this work^27^. As for the shaft washer, its inner diameter of is 35 mm and its outer diameter is 52 mm, with a thickness of 3.5 mm. The material of cage is nylon (PA66). The material of the other parts (i.e. rollers, shaft washer and housing washer) is GCr15 steel, with a surface hardness of 60 ± 1 HRC.

There were six kinds of leaves selected (Forsythia, Clausena lansiu, Ash, Purple leaf plum, Pipal and Apricot). Their corresponding vein-bionic patterns were introduced and six groups were marked as S1- S6. Each group consists of three bearings. A smooth group was used as a reference and coded as S7. So, the total number of bearings consumed in this work is 3 × 7 = 21. It should be noted that the present study complies with relevant institutional, national, and international guidelines and legislation.

**Figure 1:**
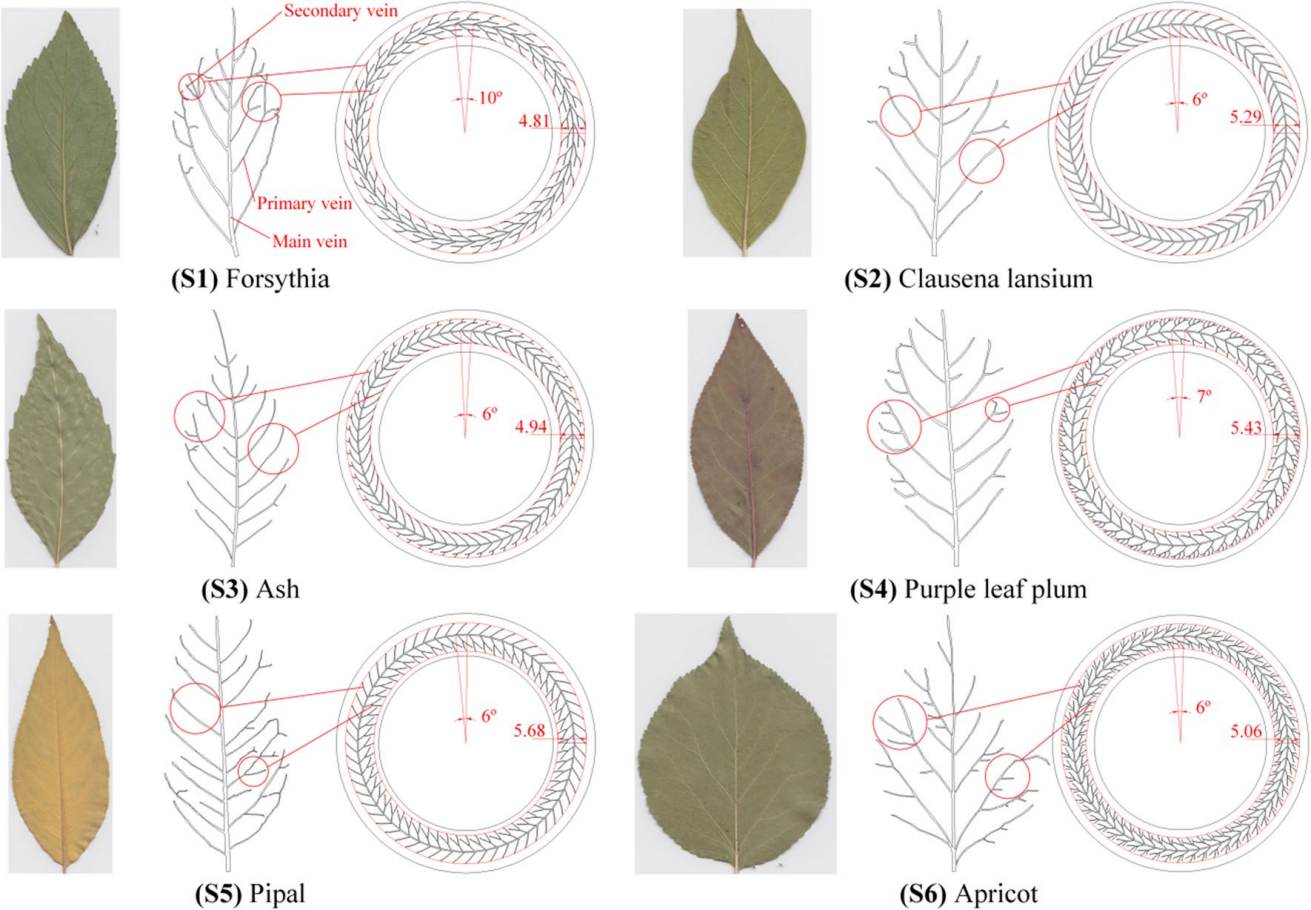
Images of leaves, captured veins and the simplified vein-bionic patterns.

**Figure 2:**
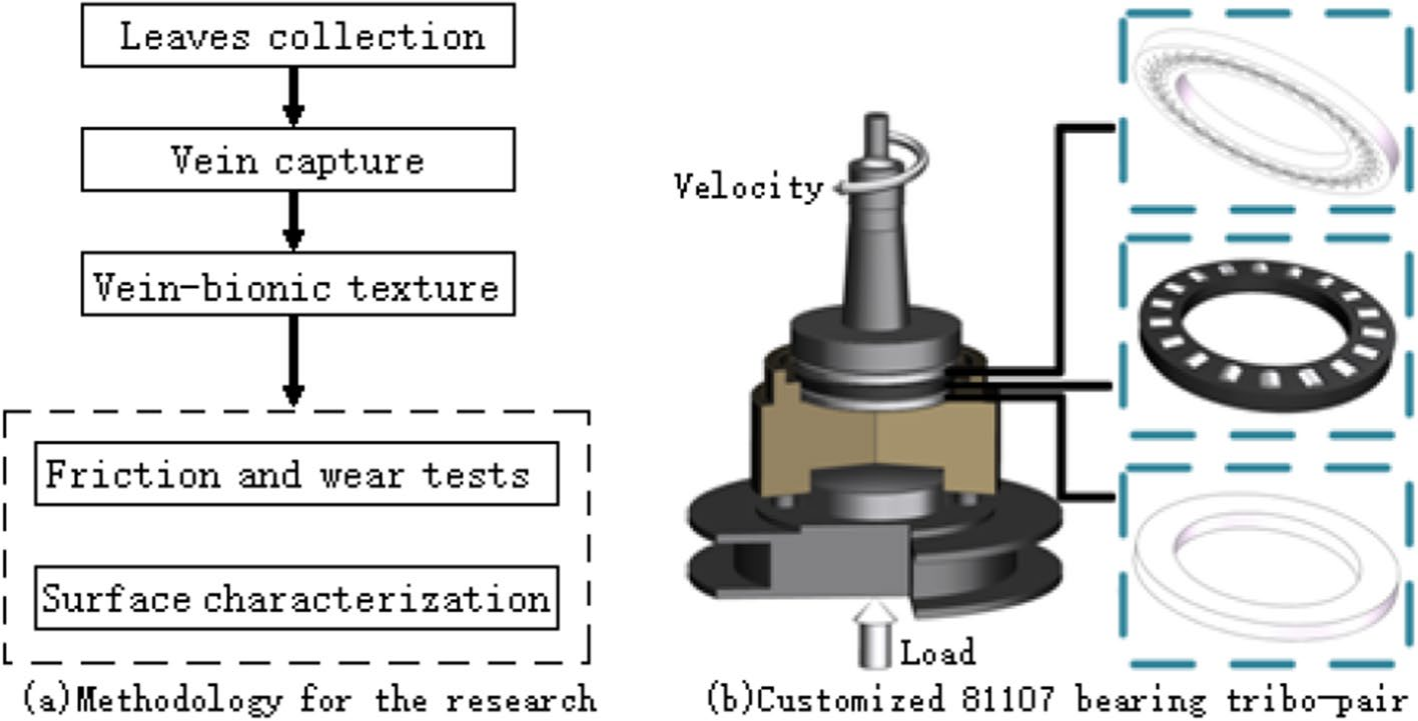
Methodology for the research and the customized 81,107 bearing tribo-pair.

**Figure 3:**
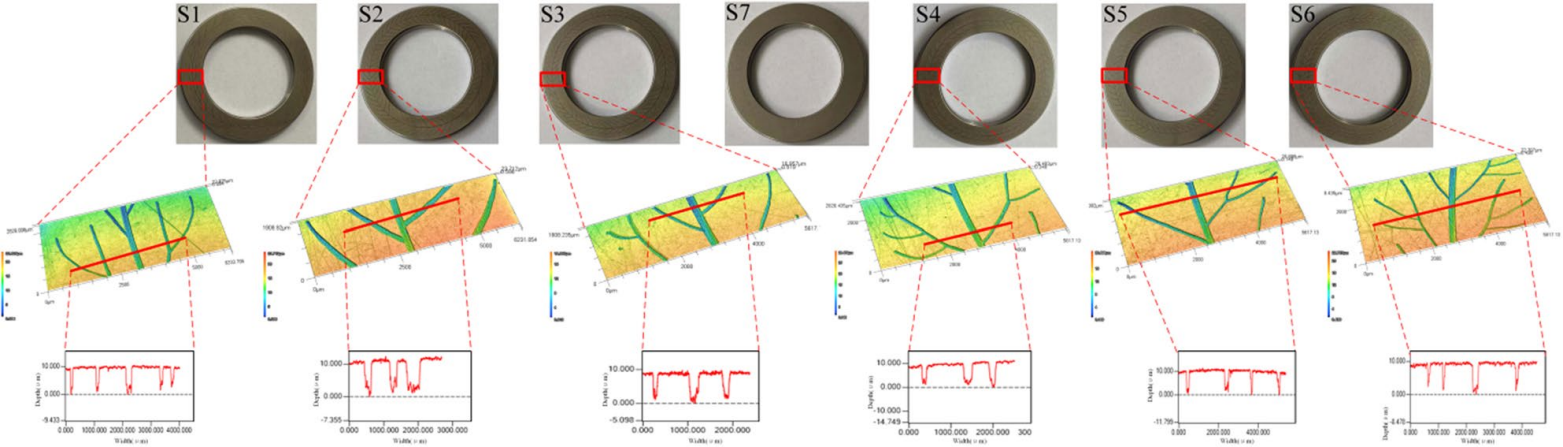
Optical images, 3D topographies and profile curves of vein-bionic textured bearings (S1–S6).

Figure 1 shows the images of leaves, captured veins and vein-bionic patterns. The research methodology is shown in Fig. 2a. The vein-bionic texture patterns were fabricated on the raceways of the shaft washers of bearings through a fiber laser marking system (PL100-30W, Saipu, China). The making parameters include: laser wavelength, 1064 nm; laser frequency, 72 kHz; laser power, 8%; scanning speed, 100 mm/s and marking times, 4. Based on repeated trials, the depth of all veins are set to 8 μm, and the width of main veins are 200 μm. The widths of primary veins and secondary veins of S1-S6 are different. Figure 3 shows the 3D images and profile curves of different vein-bionic bearings (S1–S6) after LST. According to the number of primary and secondary veins on both sides of the main vein, the texture patterns of S1, S2 and S6 are regarded as symmetrical.

### Experimental setting

Prior to each test, a drop (9 mg, a mean of twenty measurements) of commercial lubricating oil (HX7, 5W-30, Shell, bought on www.jd.com) with a dynamic viscosity of 14.45 mm^2^/s and a density of 0.8678 kg l^−1^@ 30 °C, was dripped on the raceway of the shaft washer. Through repeated tests, 9 mg is the minimum amount of oil that can ensure there is an obvious COF variation of smooth bearing and can complete the whole test. During the wear test, there was no lubricating oil supplied. In addition, the vein-bionic textured shaft washer should be polished by sandpapers (from No. 400-No. 1500) to remove the bulges formed along the edges of veins before wear test. The final line roughness of vein-bionic textured groups is about 1.1–1.3 μm, and that of smooth group is 0.693 μm.

A customized 81107 bearing tribo-pair was used to research the tribological performance of bearings under starved lubrication (see Fig. 2b). The shaft washer rotates under the driving of a servo drive system with a rotation speed of 250 r/min. The axial force is loaded by an electronic servo-cylinder and equal to 3920 N, which is determined through repeated trials. The duration of each test is 18000 s, which is determined by the maximum sampling number of test rig. The test of each group was repeated three times. The mass losses of samples were measured using an electronic balance with a precision of 0.1 mg (0.01 mg readability). Their worn surfaces were characterized by a non-contact 3D profilometer (work mode: white light and confocal laser).

## Results and discussion

### COFs and wear losses

**Figure 4:**
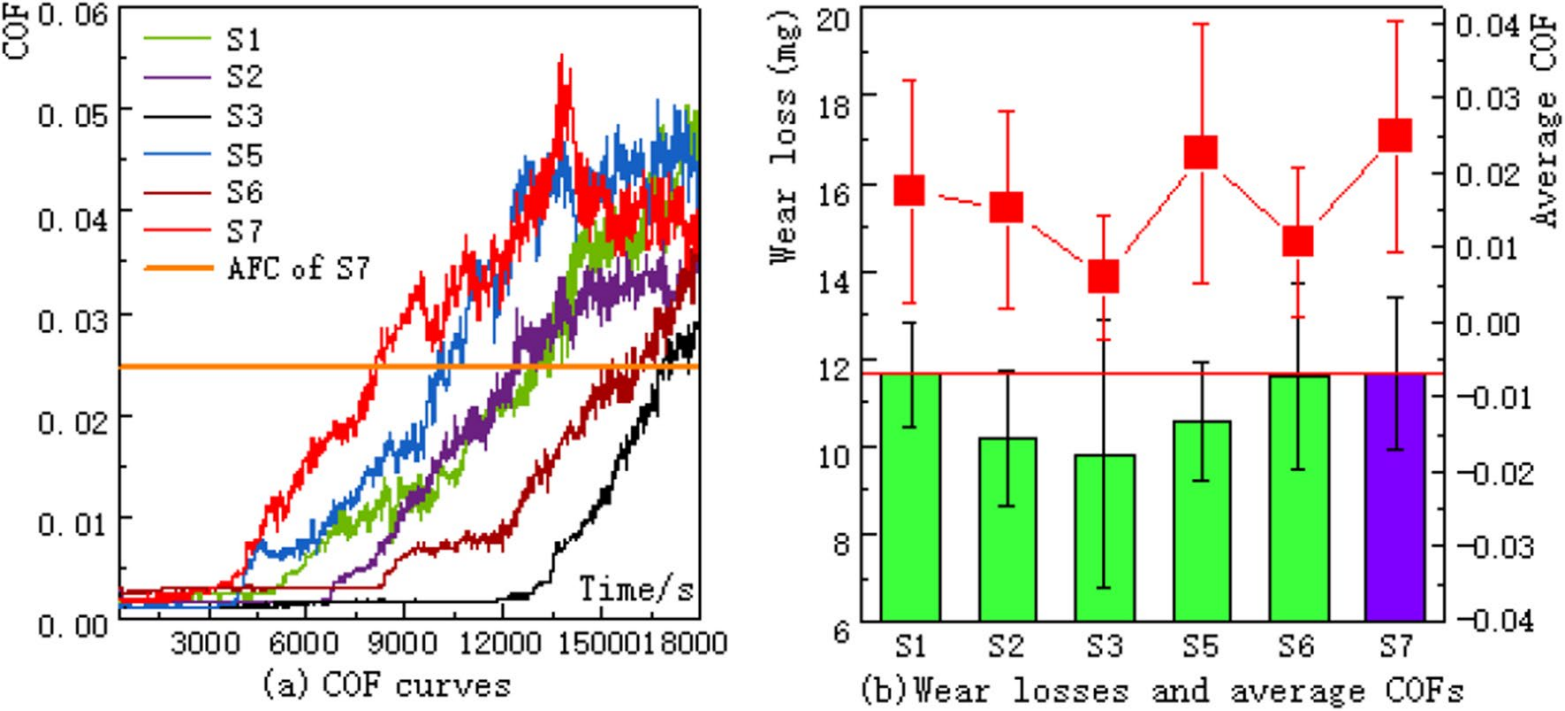
COF curves, wear losses and average COFs of groups (S1–S3, S5–S7) under starved lubrication.

The COFs, mass losses and average COFs of bearings under starved lubrication are shown in Fig. 4. Note that: The COF curve of each group is the mean value curve of three tests. All three tests of S4 failed due to the instantaneous high friction caused by cage fracture, which exceeded the safety threshold and led to the self-protective shutdown of the equipment. So, there is no data of S4 provided in the figure. As shown in Fig. 4a, there is a stable-running period of each COF curve, and then the curve will rise rapidly. All average COFs and mass losses of vein-bionic textured groups are lower than those of smooth bearings (see Fig. 4b).

### Worn surfaces

**Figure 5:**
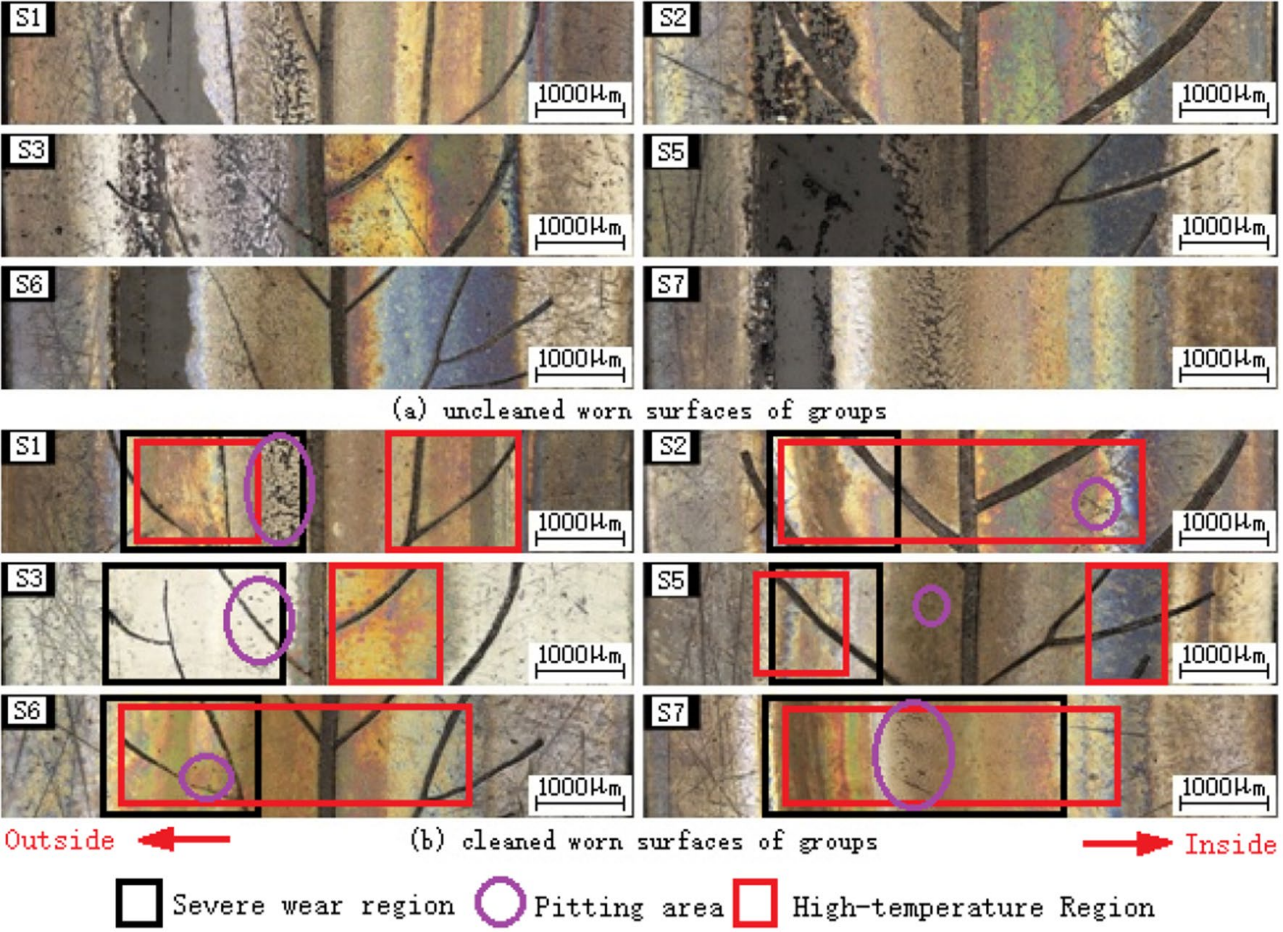
Worn surfaces of the shaft washers of groups (S1–S3, S5–S7).

**Figure 6:**
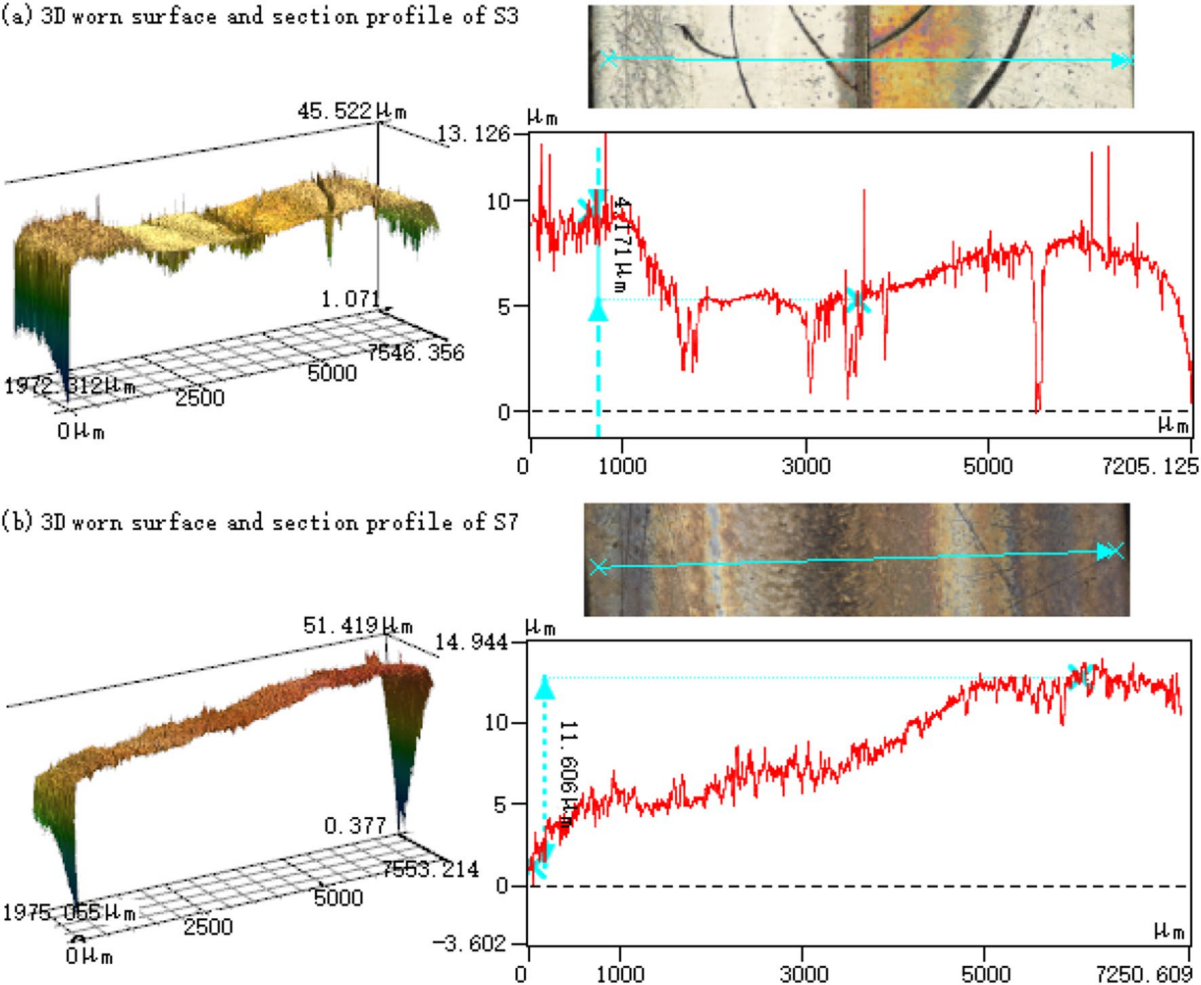
3D worn surfaces and section profiles of S3 and S7.

Figure 5 shows the worn surfaces of the shaft washers of groups (S1–S3, S5–S7) before and after cleaning. As shown in Fig. 5a, there is nylon film formed and concentrated near the outside of the raceway of each group. There are obvious abrasive wear marks on the secondary veins of S1 and S6. There is also severe fatigue pitting area near the main vein of S1. Apparently, the nylon film of S3 is much thinner than the other groups (see Fig. 5a), and its wear marks are also slight and shallow (see Figs. 5b and 6). Based on their worn surfaces, the main failure mode of all bearings is abrasive wear combined with fatigue pitting.

### Discussion

As shown in Fig. 4, the stable-running durations of all groups are sorted as: S7 < S5 < S1 < S2 < S6 < S3. The average COFs of S1–S7 are sorted as: S3 < S6 < S2 < S1 < S5 < S7. The mass losses of S1–S7 are sorted as: S3 < S2 < S5 < S1 < S6 < S7. Obviously, the average COF of S3 is the lowest among all groups and its stable-running time is the longest (13,000 s). The wear loss of S3 is also the lowest and only 9.8 mg, which is 83.77% of that of S7. In addition, the COFs of groups with symmetrical patterns (i.e. S1, S2 and S6) are significantly reduced compared to the smooth one and sorted as: S6 < S2 < S1. The mass losses of them are all smaller than that of S07 too and sorted as: S2 < S1 < S6. Specifically, the COF of S6, with two secondary veins on each side, is the smallest. This should be attributed to its more uniform lubricating film due to the larger area ratio of textured raceway during starved lubrication. The reason for the high mass losses of S1 and S6 may be due to their apparently reduced effective contact areas (ECAs). ECA equals the raceway area minus the surface area occupied by vein texture. The reduced ECAs will cause serious fatigue pitting or edge crushing and increase the metal debris left on the raceways^25,27^. This is also the reason for the serious abrasive wear marks on the raceways of S1 and S6. The wear loss of S2, without secondary vein, is the lowest. This should be attributed to its relatively high ECA among three groups.

Compared with the data of smooth group, the tribological performance (both COFs and wear losses) of those bearings with asymmetric patterns (i.e. S3 and S5) is significantly enhanced. The group S3, with one secondary vein on the outside of main vein, shows better friction reduction and anti-wear improvement than those of S5, with one secondary vein on the inside of main vein. This is because under the action of centrifugal force, the wear debris and nylon powder will move to the outside of raceway^28–30^. In this case, the secondary vein on the outside can collect and store more wear debris than the vein on the inside, and hereby reduce the thickness of the nylon film, which can be confirmed by the uncleaned worn surface of S3 in Fig. 5a. Owing to the relatively lower COF between steel and steel than that between nylon and steel, the COF and mass loss of S3 will be finally reduced^27^.

Therefore, based on the above facts, the influence of vein-bionic textures on the friction and wear properties of CRTBs can be briefly summarized as: (1) Less secondary veins are helpful to reduce wear loss and more secondary veins are helpful to reduce COF. So, to obtain good comprehensive performance of friction-reduction and wear- resistance, the quantity of secondary veins should be optimized. Furthermore, when there are too many secondary veins, i.e. S4, with three on the outside and one on the inside, the tribological behavior of bearings is significantly deteriorated and cannot even pass the wear tests. (2) Compared with the smooth and symmetrical patterns, the tribological properties of asymmetric patterns are better, and both their COFs and wear losses are reduced. 3) The final friction and wear properties of vein-bionic textured bearings under starved lubrication are mainly determined by the comprehensive effect of following factors: ECA, maximum effective volume of textures, area ratio, symmetry and quantity of secondary veins^18–20,26–30^.

## Conclusions

In this paper, the tribological properties of vein-bionic textured CRTBs inspired from six kinds of leaves (Forsythia, Clausena lansiu, Ash, Purple leaf plum, Pipal and Apricot) were investigated under starved lubrication. The results show that the symmetry of vein-bionic texture and the number of secondary veins are the key factors affecting the tribological behavior of textured bearings. In this work, compared with those of smooth group, the wear loss of S3, inspired from Ash, is reduced by 16.23% and its COF is reduced by 15.79%, showing excellent friction-reducing and wear-resistance performance.

Next, we will continue to study the friction and wear properties of more vein-bionic patterns from more kinds of leaves, and access the importance of vein texture symmetry, angle of primary vein, number of secondary veins and width variation of veins to the final comprehensive friction and wear properties.
